# Impact of Robotic-Assisted Partial Nephrectomy with Single Layer versus Double Layer Renorrhaphy on Postoperative Renal Function

**DOI:** 10.3390/curroncol31050209

**Published:** 2024-05-13

**Authors:** Hiroyuki Ito, Keita Nakane, Noriyasu Hagiwara, Makoto Kawase, Daiki Kato, Koji Iinuma, Kenichiro Ishida, Torai Enomoto, Minori Nezasa, Yuki Tobisawa, Takayasu Ito, Takuya Koie

**Affiliations:** 1 Department of Urology, Gifu University Graduate School of Medicine, Gifu 5011194, Japan; ito.hiroyuki.d8@f.gifu-u.ac.jp (H.I.); nakane.keita.k2@f.gifu-u.ac.jp (K.N.); kawase.makoto.g5@f.gifu-u.ac.jp (M.K.); kato.daiki.m2@f.gifu-u.ac.jp (D.K.); iinuma.koji.s0@f.gifu-u.ac.jp (K.I.); g.t.e.24150107@gmail.com (T.E.); tobisawa.yuki.a7@f.gifu-u.ac.jp (Y.T.); 2 JA Gifu Koseiren Seino Kosei Hospital, Gifu 5010532, Japan; hagiwara44@gmail.com; 3 Department of Urology, Matsunami General Hospital, Gifu 5016062, Japan; drken1410@gmail.com (K.I.); nezasa001@gmail.com (M.N.); 4 Center for Clinical Training and Career Development, Gifu University Graduate School of Medicine, Gifu 5011194, Japan; ito.takayasu.v9@f.gifu-u.ac.jp

**Keywords:** renal cell tumor, robot-assisted partial nephrectomy, renal function preservation, renorrhaphy, hemostasis

## Abstract

We aimed to investigate the differences in renal function between patients who underwent single inner-layer renorrhaphy (SILR) or double-layer renorrhaphy (DLR) among those with renal tumors who underwent robot-assisted partial nephrectomy (RAPN). This retrospective multicenter cohort study was conducted between November 2018 and October 2023 at two institutions and included patients who underwent RAPN. In total, 93 eligible patients who underwent RAPN were analyzed. Preoperative renal function and prevalence of chronic kidney disease were not significantly different between the two groups. Although urinary leakage was observed in three patients (5.9%) in the SILR group, there was no significant difference between the two groups regarding surgical outcomes (*p* = 0.249). Serum creatinine levels after RAPN were significantly lower in the SILR group than in the DLR group on postoperative days 1 and 365 following RAPN (*p* = 0.04). The estimated glomerular filtration rate (eGFR) was significantly lower in the DLR group than in the SILR group only on postoperative day 1; however, there was no significant difference between the two groups thereafter. Multivariate analysis showed that the method of renorrhaphy was not a predictor for maintaining renal function after RAPN even though it was associated with eGFR on postoperative day 1.

## 1. Introduction

In recent years, nephron-sparing surgery, particularly robot-assisted radical nephrectomy (RAPN), has emerged as a widely adopted treatment modality for patients with a renal mass < 7 cm in size, chronic kidney disease, or bilateral renal tumors. It is deemed technically feasible owing to its ability to better preserve renal function [1,2,3,4,5]. Although radical nephrectomy improves oncological outcomes in patients with renal cell carcinoma (RCC), its utility remains controversial as it results in the substantial loss of nephron tissue, potentially contributing to increased morbidity from chronic kidney disease, mortality from cardiovascular disease, and decreased overall survival [6,7].

RAPN is a surgical technique aimed at achieving a “trifecta” outcome, defined as (I) negative surgical margins, (II) maximum preservation of normal renal parenchyma, and (III) complete recovery without perioperative complications [1,3]. Similarly, renorrhaphy is a key process in achieving adequate hemostasis and renal reconstruction within an acceptable warm ischemic time during RAPN [6]. Four surgical techniques have been proposed for performing renorrhaphy: (1) single-layer lateral cortical renorrhaphy [8], (2) single inner-layer renorrhaphy (SILR) with early unclamping [5], (3) double-layer renorrhaphy (DLR) [9], and (4) coagulation of the base following the removal of the renal tumor and sutureless renal parenchyma [1]. Nevertheless, parenchymal reconstruction by renorrhaphy may damage the renal artery branches and affect perirenal blood flow, resulting in irreversible ischemic changes in the remaining normal renal parenchyma [6]. This may result in increased parenchymal volume loss, decreased renal function, and pseudoaneurysm development [10,11,12]. Although renal function at the renorrhaphy site is likely to be impaired, the precise relationship between the renorrhaphy method and evolution of postoperative renal function remains unclear. In addition, several reports recommend sutureless approach of the renal parenchyma to preserve renal function after partial nephrectomy (PN) as much as possible [13,14]. Although this procedure is expected to have favorable outcome in preserving renal function, “trifecta” could not be achieved in all patients at many institutions [13,14].

In this study, we aimed to investigate the differences in renal function between patients who underwent SILR and DLR in those who were monitored for at least 1 year following RAPN.

## 2. Materials and Methods

### 2.1. Patient Population

The Institutional Review Board of Gifu University authorized this study (approval number: 2023-260). Given the retrospective nature of the study, written informed consent was not obtained from all enrolled patients, and consent was obtained through opt-out procedures. In Japan, retrospective and observational studies necessitate disclosure of research information, including existing materials, and this study was conducted in accordance with the provisions of the ethics committee and ethical guidelines. Details of this study, which were prepared in Japanese only, can be accessed at https://www.med.gifu-u.ac.jp/ (accessed on 1 March 2024).

This retrospective multicenter cohort study was conducted between November 2018 and October 2023 at Gifu University Hospital and Matsunami General Hospital and included patients with renal tumors who underwent RAPN with the supervision of a single expert surgeon (T.K.). All enrolled patients with renal tumors underwent computed tomography (CT) of the chest, abdomen, and pelvis to assess the baseline characteristics prior to RAPN. Tumor staging was the American Joint Committee on Cancer Staging Manual [13]. Pretreatment clinical data of all enrolled patients were collected, including age, sex, body mass index (BMI), Eastern Cooperative Oncology Group performance status (ECOG-PS) score [15], smoking history, comorbidities, clinical T stage, tumor size and location, neutrophil count, lymphocyte count, neutrophil-to-lymphocyte ratio (NLR), creatinine (Cr) level, estimated glomerular filtration rate (eGFR), and C-reactive protein (CRP) level. The eGFR was calculated using the Modification of Diet in Renal Disease 2 equation and further modified for Japanese patients using the equation proposed by the Japanese Society of Nephrology: eGFR = 1.94 × serum Cr (mg/dL) − 1.094 × age × (0. 739, for women) [16]. Tumor complexity was defined using the R.E.N.A.L. nephrometry score, comprising (R)adius (tumor size as maximal diameter), (E)xophytic/endophytic properties of the tumor, (N)earness of the tumor deepest portion to the collecting system or sinus, (A)nterior/posterior descriptor, and (L)ocation relative to the polar line [17].

### 2.2. Surgical Procedure

A da Vinci X or Xi system (Intuitive Surgical, Sunnyvale, CA, USA) was used to perform RAPN. In this study, all RAPN procedures were carried out by skilled surgeons with experience in at least 20 relevant cases. The surgery commenced with the patient placed in the lateral decubitus position, and three robotic ports, a camera port, and one or two assistant ports were placed after insufflation. Depending on the size and location of the renal tumor, the surgeon opted for either the transperitoneal or retroperitoneal approach. Ultrasound guidance was employed to confirm the size and depth of the tumor, and the line of the resection of the renal tumor was determined. The main renal artery was completely clamped using laparoscopic bulldog clamps, whereas the venous vascular system remained untouched. After reaching the peritumoral parenchyma, the tumor was bluntly dissected along with the fibers of the renal parenchyma and surgically removed with thin margins, preserving as much normal parenchyma as possible. During tumor removal, the assistant utilized suction and performed soft coagulation using a ball-shaped electrode (Valleylab™ FT10; Covidien, New Haven, CT, USA) to control bleeding from the renal parenchyma.

In the SILR technique, single-layer inner running sutures were employed to selectively repair the open collecting system, micro blood vessels, and renal sinus using a 15 cm 3-0 V-Loc™ CV-23 (Covidien, New Haven, CT, USA). The bulldog clamp was then removed, and Tacosil^®^ (CSL Behring, Tokyo, Japan) was placed in the resection bed as an absorbable hemostatic material. Finally, the kidney surface was covered with perineal fat using 2-0 or 3-0 V-Loc™. In the DLR technique, Tacosil was applied to the resection surface after performing SILR. Subsequently, the renal parenchyma was sutured at 2 cm intervals using 2-0 V-Loc and anchored with Hem-o-loc clips^®^ (Teleflex, Morrisville, NC, USA). The kidney surface was also covered with perineal fat using 2-0 or 3-0 V-Loc™. Contrast-enhanced CT was performed in all patients who underwent RAPN between 7 and 10 days postoperatively to assess for surgery-related complications such as rebleeding, pseudoaneurysm, and urinary leakage.

All patients who underwent RAPN were evaluated for renal function including eGFR every 3 months, and CT was performed every year to check for metastasis or recurrence. Inclusion criteria for this study were patients who could be followed up for at least 1 year after RAPN and whose renal function was evaluated. The enrolled patients were divided into two groups based on the renorrhaphy technique employed: the SILR and DLR groups. The method for performing renorrhaphy was selected based on the size, depth, and location of the renal tumor as well as the surgeon’s preference. The following data were also collected as factors related to surgery: renorrhaphy method, console time, warm ischemic time (WIT), whether the collecting system was opened, estimated blood loss (EBL), blood transfusion, length of hospital stay (LOS), pathologic T classification, histological type, and resection margin status. To confirm the quality of surgery, we also evaluated whether the trifecta criteria (which comprised (1) negative surgical margins, (2) WIT within 25 min, and (3) the absence of perioperative complications) and the pentafecta criteria (which, in addition to the trifecta criteria, included (4) the maintenance of >90% postoperative renal function and (5) the absence of chronic kidney disease deterioration 1 year after surgery) were met [8,18]. The evaluation of renal function deterioration after RAPN was performed according to the method reported by Simon et al. [19].

### 2.3. Statistical Analysis

The primary endpoint was post-RAPN recovery of renal function based on the renorrhaphy method used. The secondary endpoints were perioperative outcomes and chronological changes in inflammation-related factors, including CRP level and NLR, following RAPN between the two groups. We analyzed the collected data using JMP Pro 17 software (SAS Institute Inc., Cary, NC, USA). Continuous and categorical variables between the two groups were compared using the Mann–Whitney U test or Kruskal–Wallis test. The follow-up period was defined as the period from the date of RAPN implementation to the date of the last follow-up survey. Multivariate analysis was performed using the linear logistic regression analysis to examine the preoperative and surgery-related factors that affect renal function after RAPN. By contrast, the present study did not enroll a sufficient number of patients to be correctly evaluated by Ridge or Lasso regression. Based on several previously reported studies, six variables were selected for univariate and multivariate analyses in order to prevent overfitting [2,20]. Dependent variables for creatinine were mg/dL and eGFR was mL/min/1.73 m^2^. Two-tailed *p*-values were calculated, and the statistical significance level was set at a *p*-value < 0.05.

## 3. Results

### 3.1. Characteristics of the Patient before RAPN

Table 1 shows the patients’ preoperative characteristics. A total of 26 patients who were not observed for ≥365 days, 1 patient who underwent intraoperative conversion to radical nephrectomy, and 1 patient who had missing data were excluded from the study. Ultimately, only 93 patients who underwent RAPN met the inclusion criteria.

The median and interquartile range (IQR) of age, BMI, tumor size, and R.E.N.A.L nephrometry score in all patients were 66 years (55–73 years), 23.8 (21.6–26.9), 20 mm (13.8–30.0 mm), and 6 (5–7), respectively. Although patients in the SILR group were significantly older and had a significantly shorter follow-up period, no significant differences were found in other factors between the two groups.

### 3.2. Surgical and Pathological Outcomes after RAPN (Table 2)

None of the enrolled participants died of renal tumors or had metastases at the end of the follow-up period. In all patients, the median console time, WIT, and EBL was 115 min (IQR, 93–142 min), 19 min (IQR, 14–23 min), and 10 mL (IQR, 5–50 mL), respectively. No significant differences were observed between the two groups in any of the variables.

With regard to surgery-related complications, none of the patients had perioperative complications such as postoperative bleeding, pseudoaneurysm, ileus, or oliguria. Although three patients (5.9%) in the SILR group developed urinary leakage as a surgery-related complication, there were no incidences of this complication in the DLR group (*p* = 0.249). However, all three patients were successfully cured with conservative treatment.

**Table 2:** Comparison of perioperative and pathological outcomes in patients who underwent robot-assisted partial nephrectomy.

Covariates	SILR Group (*n* = 51)	DLR Group (*n* = 42)	*p*-Value
Approach (number, %)			0.202
Retroperitoneal	23 (45.1)	13 (31.0)	
Transperitoneal	28 (54.9)	29 (69.0)	
Console time (min, median, IQR)	115.0 (90.5–145.5)	108.5 (95.0–136.3)	0.889
Warm ischemia time (min, median, IQR)	17.5 (13.0–21.8)	19.0 (17.0–23.8)	0.206
Opening of renal pelvis (number, %)	10 (19.6)	8 (19.0)	>0.999
Blood loss (mL, median, IQR)	20.0 (5.0–50.0)	5.0 (5.0–30.0)	0.101
Blood transfusion (number, %)	1 (2.0)	0 (0.0)	>0.999
LOS (days, median, IQR)	8.0 (7.0–9.5)	8.0 (7.0–9.0)	0.361
Pathological T stage (number, %)			>0.999
1a	42 (91.3)	33 (91.7)	
1b	3 (6.5)	2 (5.6)	
3a	1 (2.2)	1 (2.8)	
Pathological diagnosis (number, %)			0.149
Clear cell	40 (78.4)	30 (71.4)	
Papillary	3 (5.9)	3 (7.1)	
Chromophobe	2 (3.9)	3 (7.1)	
Angiomyolipoma	2 (3.9)	6 (14.3)	
Others	4 (7.8)	0 (0.0)	
Positive surgical margin (number, %)	1 (2.1)	2 (4.9)	0.593
Decrease in eGFR by ≥10% up to 365 days postoperatively (number, %)	13 (35.1)	13 (43.3)	0.615
Worsening of CKD stage after 365 days (number, %)	9 (24.3)	7 (23.3)	>0.999
Worsening to CKD stage ≥4 at 365 days (number, %)	1 (2.7)	1 (3.3)	>0.999
Trifecta (number, %)	37 (78.7)	32 (78.0)	>0.999
Pentafecta (number, %)	18 (52.9)	12 (44.4)	0.609

SILR, single inner-layer renorrhaphy; DLT, double-layer renorrhaphy; IQR, interquartile range; LOS, length of hospital stay; CKD, chronic kidney disease.

### 3.3. Chronological Changes in Renal Function after Surgery

**Figure 1:**
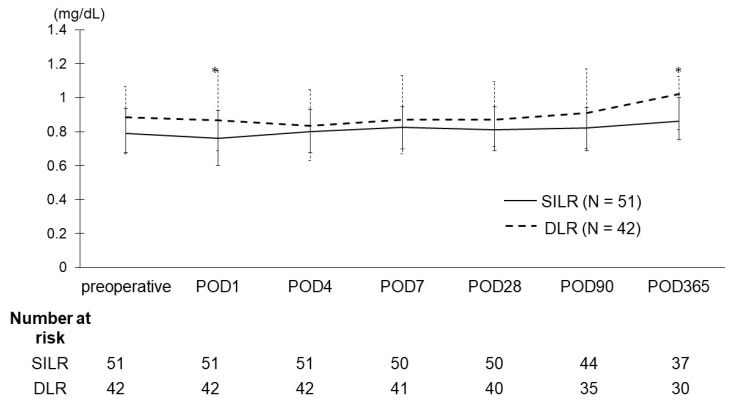
The trends and interquartile range of serum creatinine levels after robot-assisted partial nephrectomy (RAPN). The creatinine values on postoperative days 1 and 365 were significantly lower in the SILR group compared with in the DLR group (0.76 mg/dL vs. 0.87 mg/dL, *p* = 0.04; 0.86 mg/dL vs. 1.02 mg/dL, *p* = 0.04). However, no significant differences were observed between the two groups at 4, 7, 28, and 90 days postoperatively. SILR, single-inner layer renorrhaphy; DLR, double-layer renorrhaphy.

Figure 1 illustrates the chronological changes in Cr levels after RAPN. On days 1 and 365 following RAPN, serum Cr levels were significantly lower in the SILR group compared with in the DLR group (0.76 mg/dL vs. 0.87 mg/dL, *p* = 0.04; 0.86 mg/dL vs. 1.02 mg/dL, *p* = 0.04). Throughout the follow-up period, a tendency toward higher Cr levels was observed in the DLR group compared with in the SILR group.

**Figure 2:**
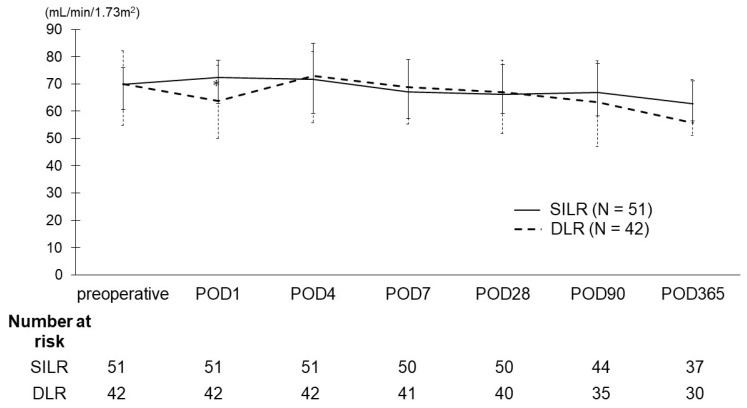
Changes and interquartile range of estimated glomerular filtration rate (eGFR) after robot-assisted partial nephrectomy (RAPN). The eGFR on postoperative day 1 was significantly lower in the DLR group compared with in the SILR group (72.4 mL/min/1.73 m^2^ vs. 63.8 mL/min/1.73 m^2^, *p* = 0.04). However, no significant differences were observed between the two groups at 4, 7, 28, 90, and 365 days after RAPN. SILR, single-inner layer renorrhaphy; DLR, double-layer renorrhaphy.

Figure 2 indicates eGFR trends after RAPN. The eGFR significantly decreased after RAPN on day 1 in the DLR group compared with in the SILR group, whereas no significant difference was noted between the two groups thereafter.

Both the univariate and multivariate analyses identified preoperative renal function emerged as a significant factor influencing the change in postoperative renal function for Cr level and eGFR, whereas renorrhaphy was an independent predictor for influencing the change in eGFR on the first postoperative day only (Table 3).

## 4. Discussion

The most important factors for performing RAPN include achieving complete oncological control, preserving maximal renal function, and minimizing complications. The WIT and amount of healthy renal parenchyma resected are considered key determinants of renal function and renal parenchyma volume loss following RAPN [21]. Among these factors, the decline in renal function is believed to be primarily associated with the reduction in renal volume rather than the WIT [8]. With regard to renorrhaphy, which is performed to achieve hemostasis, the amount of healthy renal tissue incised to remove the tumor can vary [22,23]. Therefore, the amount of normal renal parenchyma, which contains a large number of vascular vessels, and the method of renorrhaphy can be major factors in determining the long-term preservation of renal function [22,23]. Furthermore, the selection of renorrhaphy technique is highly dependent on the surgeon’s experience and approach to tumor removal [8]. Although the primary objective of renorrhaphy is to ensure hemostasis and reduce postoperative complications, the preservation of postoperative renal function remains controversial.

In this study, no significant differences were observed in the surgical outcomes, rates of achieving trifecta and pentafecta, or perioperative complications between the two groups. In terms of perioperative complications, only three patients experienced urinary leakage in the SILR group, and postoperative bleeding or pseudoaneurysm did not occur in either group. The rates of achieving trifecta and pentafecta were also relatively high compared with those reported in previous studies, suggesting that the quality of surgery was acceptable [24,25]. In terms of the renorrhaphy method, no significant differences were noted in LOS, renal function, or incidence of postoperative complications, although SILR contributed to a reduction in operative time and WIT [26]. The operative time, console time, WIT, and LOS did not significantly differ according to the renorrhaphy method used; however, the incidence of urinary leakage or urinoma was significantly higher in the SILR group compared with in the DLR group [27]. One of the primary concerns related to SILR is the occurrence of postoperative complications, especially bleeding and urinary leakage [28]. However, another meta-analysis showed no significant difference in the incidence of postoperative complications between SILR and DLR [28]. Hemostatic running sutures frequently performed during RAPN, especially deep in the renal medulla, may encircle the branches of vessels traveling in proximity [29]. Therefore, SILR is thought to significantly reduce the incidence of pseudoaneurysms as suturing the renal parenchyma during renorrhaphy helps prevent injury to the renal vessels [28]. Furthermore, repairing all transected vessels by carefully performing clipping or oversewing during DLR remains technically challenging [8]. Therefore, we considered SILR a technically suitable procedure owing to the comparable perioperative outcomes and incidence of complications in comparison with DLR.

Currently, the primary goal of DLR is to employ a two-layered approach: the first layer closes the collecting system and ligates bleeding vessels, whereas the second layer closes the renal cortex and parenchyma [30]. Postoperative reductions in renal volume are attributed to tumor resection rather than renal reconstruction [21]. Therefore, ischemia time and the amount of resected healthy renal parenchyma are considered the key factors affecting renal function and volume reduction following RAPN [21]. However, only a few studies have evaluated the effects of renorrhaphy methods on renal function [10,21,30]. One study showed a significant difference in residual renal volume reduction, with a 17% decrease in the DLR group and a 9% decrease in the SILR group (*p* = 0.003) [17]. Additionally, an eGFR comparison between the two groups showed an 8.8% decrease in the DLR group and a 4.4% decrease in the SILR group 4 months after surgery [20]. In a study of 118 and 34 patients who underwent DLR and SILR, respectively, the reduction in renal parenchymal volume was significantly higher in the DLR group (15.6%) compared with in the SILR group (3.8%) (*p* = 0.03) [10]. Furthermore, multivariate analysis revealed that renorrhaphy was an independent predictor of renal volume loss after RAPN (*p* < 0.01) [10]. Another study demonstrated that patients who underwent SILR reported a shorter WIT and lower rate of worsening eGFR and renal volume at 4 months postoperatively compared with those who underwent DLR [27]. Renorrhaphy and the resulting traumatic lesions may be more crucial than the amount of tumor resected in predicting residual renal function after RAPN [6]. In this study, a transient decrease in eGFR was observed in the DLR group; however, the subsequent changes were similar in both groups. In the multivariate analysis, preoperative renal function was a useful predictor of maintenance of good postoperative renal function, although the renorrhaphy method showed no statistical difference. Our DLR procedure may minimize the impact on the remaining renal parenchyma owing to its rough suturing. However, considering that the Cr levels remained lower in the SILR group compared with in the DLR group, although the difference was not significant, performing DLR for all patients undergoing RAPN may not be necessary.

Regarding the possibility that WIT may have an important influence on renal function after RAPN, the so-called off-clamp surgery, in which the renal artery is not clamped, should be considered. Bertolo et al. [20] reported on the surgical results of RAPN with SILR and off-clamp, with no positive margins and minimal postoperative renal function loss. Simone et al. [31] divided 1073 patients who underwent partial nephrectomy (PN) into off-clamp PN and on-clamp PN groups by propensity score matching (3:1) and evaluated long-term renal function after surgery in both groups. The results showed that renal function was maintained longer in the off-clamp group (58% vs. 4%; *p* = 0.02) and the number of patients with stage ≥3b chronic kidney disease was significantly lower in this group than in its counterpart (*p* < 0.001) [29]. In a multivariate analysis, off-clamping was also an independent predictor of long-term maintenance of renal function after PN [31]. Therefore, it may need to be considered in patients selected for RAPN with off-clamping, although all patients in this study underwent RAPN with clamping of the renal artery [32].

Several limitations should be considered in this study. First, the small number of enrolled patients and retrospective nature of the study may introduce bias. Second, the results should be interpreted with caution because the renorrhaphy method was not randomly assigned but rather determined based on the tumor location and surgeon preference. Third, we did not evaluate the changes in residual function or renal parenchymal volume after RAPN using a renogram. In addition, postoperative status of comorbidities such as hypertension and diabetes mellitus were not assessed. Therefore, the reason for patients with worsening renal function after RAPN could not be clearly answered. Finally, the follow-up period of the study was not long enough to allow for any mention of oncological outcomes or long-term changes in renal function.

## 5. Conclusions

In this study, we compared the post-RAPN outcomes of SILR and DLR. The different renorrhaphy methods had no effect on long-term eGFR; however, significant differences were observed in Cr levels on the day after surgery and on day 365. Therefore, a large prospective study is required to clarify the effects of renorrhaphy methods on renal function.

## Figures and Tables

**Table 1:** Preoperative patient characteristics.

Covariates	SILR Group (*n* = 51)	DLR Group (*n* = 42)	*p*-Value
Age (year, median, IQR)	69 (60.5–74.0)	61 (51.0–70.5)	0.002
Sex (number, %)			0.377
Male	33 (64.7)	31 (73.8)	
Female	18 (35.3)	11 (26.2)	
Body mass index (kg/m^2^, median, IQR)	23.9 (21.8–26.5)	23.6 (21.2–27.3)	0.740
ECOG-PS (number, %)			0.091
0	46 (90.2)	32 (76.2)	
1	5 (9.8)	10 (23.8)	
Smoking history (number, %)	22 (43.1)	15 (35.7)	0.523
Comorbidity			
Hypertension (number, %)	29 (56.9)	21 (50.0)	0.537
Diabetes (number, %)	13 (25.5)	11 (26.2)	>0.999
Tumor side (number, %)			0.678
Right	26 (51.0)	19 (45.2)	
Left	25 (49.0)	23 (54.8)	
Clinical T stage (number, %)			>0.999
1a	48 (94.1)	39 (92.9)	
1b	3 (5.9)	3 (7.1)	
Tumor size (mm, median, IQR)	23.0 (15.5–32.5)	22.5 (18.0–30.8)	0.917
R.E.N.A.L nephrometry score (number, %)			0.420
4–6	27 (52.9)	27 (64.3)	
7–9	22 (43.1)	15 (35.7)	
10–12	2 (3.9)	0 (0.0)	
Creatinine (mg/dL, median, IQR)	0.79 (0.68–0.94)	0.89 (0.67–1.07)	0.144
eGFR (mL/min/1.73 m^2^, median, IQR)	69.8 (60.6–76.0)	69.9 (54.7–82.0)	0.832
Preoperative CKD stage (number, %)			
CKD stage 1	4 (7.8)	4 (9.5)	0.245
CKD stage 2	36 (70.6)	21 (50.0)	
CKD stage 3a	8 (15.7)	12 (28.6)	
CKD stage 3b	3 (5.9)	4 (9.5)	
CKD stage 4	0 (0.0)	1 (2.4)	
CKD stage 5	0 (0.0)	0 (0.0)	
Follow-up period (months, median, IQR)	18 (12.0–30.0)	35 (8.3–46.5)	0.025

SILR, single inner-layer renorrhaphy; DLT, double-layer renorrhaphy; IQR, interquartile range; ECOG-PS, The Eastern Cooperative Oncology Group Performance Status; eGFR, estimated glomerular filtration rate; CKD, chronic kidney disease.

**Table 3:** Univariate and multivariate analysis of renal function after RAPN.

(**a**) Univariate and multivariate analysis of creatinine levels on the first postoperative day								
**Variables**	**Univariable Analysis**				**Multivariable Analysis**			
	**β** **(95% CI)**	**SE**	**t**	***p*-Value**	**β** **(95% CI)**	**SE**	**t**	***p*-Value**
Age	0.0027 (−0.0031–0.0085)	0.0029	0.919	0.360	−0.0022 (−0.0044–0.0001)	0.0011	−1.914	0.059
Preoperative creatinine	1.0350 (0.952–1.119)	0.0420	24.677	<0.001	1.0152 (0.922–1.101)	0.0470	21.565	<0.001
R.E.N.A.L nephrometry score	0.0159 (−0.0248–0.0566)	0.0205	0.774	0.600	0.0057 (−0.0104–0.0217)	0.0081	0.670	0.486
Tumor size	0.0071 (0.0010–0.0132)	0.0031	2.316	0.345	−0.0014 (−0.0040–0.0012)	0.0013	−1.070	0.288
Warm ischemic time	0.0028 (−0.0050–0.0107)	0.0040	0.714	0.166	0.0013 (−0.0040–0.0012)	0.0018	0.708	0.481
The method of renorrhaphy	0.1360 (0.0162–0.2550)	0.0600	2.260	0.199	0.0169 (−0.0357–0.0695)	0.0264	0.638	0.526
(**b**) Univariate and multivariate analysis of creatinine levels at 365 days after surgery								
**Variables**	**Univariable Analysis**				**Multivariable Analysis**			
	**β** **(95% CI)**	**SE**	**t**	***p*-Value**	**β** **(95% CI)**	**SE**	**t**	***p*-Value**
Age	0.0054 (−0.0015–0.0124)	0.0035	1.560	0.124	0.0005 (−0.0025–0.0035)	0.0015	0.342	0.733
Preoperative creatinine	1.1420 (1.0222–1.2610)	0.0598	19.086	<0.001	1.1550 (1.0155–1.294)	0.0695	16.613	<0.001
R.E.N.A.L nephrometry score	0.0361 (−0.0155–0.0879)	0.0258	1.397	0.167	0.0095 (−0.0133–0.0322)	0.0114	0.832	0.409
Tumor size	0.0102 (0.00223–0.0181)	0.0040	2.559	0.013	−0.0029 (−0.0071–0.0013)	0.0021	−1.366	0.177
Warm ischemic time	0.0058 (−0.00416–0.0156)	0.0050	1.157	0.251	0.0031 (−0.0017–0.0080)	0.0024	1.307	0.196
The method of renorrhaphy	0.1460 (−0.0123–0.3040)	0.0791	1.841	0.070	0.0105 (−0.0604–0.0814)	0.0354	0.297	0.768
(**c**) Univariate and multivariate analysis of estimated glomerular filtration rate on postoperative day 1								
**Variables**	**Univariable Analysis**				**Multivariable Analysis**			
	**β** **(95% CI)**	**SE**	**t**	***p*-Value**	**β** **(95% CI)**	**SE**	**t**	***p*-Value**
Age	−0.282 (−0.595–0.031)	0.157	−1.792	0.076	0.209 (−0.002–0.420)	0.106	1.974	0.052
Preoperative eGFR	0.880 (0.748–1.012)	0.0664	13.258	<0.001	0.909 (0.762–1.055)	0.0738	12.312	<0.001
R.E.N.A.L nephrometry score	−1.240 (−3.461–0.981)	1.112	−1.109	0.270	−0.093 (−1.457–1.272)	0.686	−0.135	0.893
Tumor size	−0.452 (−0.781–−0.123)	0.166	−2.730	0.0076	−0.142 (−0.364–0.080)	0.111	−1.274	0.206
Warm ischemic time	−0.244 (−0.698–0.211)	0.229	−1.064	0.290	0.039 (−0.266–0.344)	0.153	0.253	0.801
The method of renorrhaphy	−0.836 (−15.264–−1.452)	3.477	−2.404	0.018	−5.368 (−9.790–−0.947)	2.223	−2.414	0.018

β, partial regression coefficient; SE, standard error; CI, confidence interval. eGFR, estimated glomerular filtration rate.

## Data Availability

All data and materials are made available in this paper.
